# Assessing batch effects of genotype calling algorithm BRLMM for the Affymetrix GeneChip Human Mapping 500 K array set using 270 HapMap samples

**DOI:** 10.1186/1471-2105-9-S9-S17

**Published:** 2008-08-12

**Authors:** Huixiao Hong, Zhenqiang Su, Weigong Ge, Leming Shi, Roger Perkins, Hong Fang, Joshua Xu, James J Chen, Tao Han, Jim Kaput, James C Fuscoe, Weida Tong

**Affiliations:** 1 Division of Systems Toxicology, National Center for Toxicological Research, US Food and Drug Administration, 3900 NCTR Road, Jefferson, AR 72079, USA; 2 Z-Tech Corp, an ICF International Company at National Center for Toxicological Research, US Food and Drug Administration, 3900 NCTR Road, Jefferson, AR 72079, USA; 3 Division of Personalized Nutrition and Medicine, National Center for Toxicological Research, US Food and Drug Administration, 3900 NCTR Road, Jefferson, AR 72079, USA

## Abstract

**Background:**

Genome-wide association studies (GWAS) aim to identify genetic variants (usually single nucleotide polymorphisms [SNPs]) across the entire human genome that are associated with phenotypic traits such as disease status and drug response. Highly accurate and reproducible genotype calling are paramount since errors introduced by calling algorithms can lead to inflation of false associations between genotype and phenotype. Most genotype calling algorithms currently used for GWAS are based on multiple arrays. Because hundreds of gigabytes (GB) of raw data are generated from a GWAS, the samples are typically partitioned into batches containing subsets of the entire dataset for genotype calling. High call rates and accuracies have been achieved. However, the effects of batch size (i.e., number of chips analyzed together) and of batch composition (i.e., the choice of chips in a batch) on call rate and accuracy as well as the propagation of the effects into significantly associated SNPs identified have not been investigated. In this paper, we analyzed both the batch size and batch composition for effects on the genotype calling algorithm BRLMM using raw data of 270 HapMap samples analyzed with the Affymetrix Human Mapping 500 K array set.

**Results:**

Using data from 270 HapMap samples interrogated with the Affymetrix Human Mapping 500 K array set, three different batch sizes and three different batch compositions were used for genotyping using the BRLMM algorithm. Comparative analysis of the calling results and the corresponding lists of significant SNPs identified through association analysis revealed that both batch size and composition affected genotype calling results and significantly associated SNPs. Batch size and batch composition effects were more severe on samples and SNPs with lower call rates than ones with higher call rates, and on heterozygous genotype calls compared to homozygous genotype calls.

**Conclusion:**

Batch size and composition affect the genotype calling results in GWAS using BRLMM. The larger the differences in batch sizes, the larger the effect. The more homogenous the samples in the batches, the more consistent the genotype calls. The inconsistency propagates to the lists of significantly associated SNPs identified in downstream association analysis. Thus, uniform and large batch sizes should be used to make genotype calls for GWAS. In addition, samples of high homogeneity should be placed into the same batch.

## Background

Genome-wide association studies (GWAS) aim to identify genetic variants of single nucleotide polymorphisms (SNPs) across the entire human genome that are associated with phenotypic traits, such as disease status and drug response. The International HapMap project determined genotypes of over 3.1 million common SNPs in human populations and computationally assembled them into a genome-wide map of SNP-tagged haplotypes [1,2]. Concurrently, high-throughput SNP genotyping technology advanced to enable simultaneous genotyping of hundreds of thousands of SNPs. These advances combine to make GWAS a feasible and a promising research field for associating genotypes with various disease susceptibilities and health outcomes. Recently, GWAS was successfully applied to identify common genetic variants associated with a variety of phenotypes [3-31]. Many of these studies used the Affymetrix GeneChip Human Mapping 500 K array set [5,6,11]. The genomic DNA for one of the arrays is cleaved with the *Nsp I *restriction enzyme and ~262,000 SNPs are interrogated. The second chip uses *Sty I *– cleaved genomic DNA and ~238,000 SNPs are analyzed. Genotypes from Affymetrix GeneChip Human Mapping 500 K array set data are usually determined by the calling algorithm BRLMM [32] embedded in Affymetrix software packages. Algorithms developed by other laboratories such as PLASQ [33], GEL [34], CRLMM [35], SNiPer-HD [36], MAMS [37], and CHIAMO [11] are also utilized.

The MPAM algorithm was developed for analysis of raw data (i.e., the CEL files) from the first generation of Affymetrix Mapping 10 K array and is based on clustering of chips for each SNP by modified partitioning around medoids [38]. MPAM was error prone for SNPs with missing genotype groups or low minor allele frequency, a problem more pronounced on the second generation of Affymetrix Mapping 100 K array. This prompted Affymetrix to develop a new dynamic model based calling algorithm called DM for Mapping 100 K array data [39]. DM is a single-chip calling algorithm and usually calls genotypes with high overall call rate and accuracy. However, the algorithm exhibited a higher misclassification rate for heterozygous genotypes than for homozygous genotypes. To improve data analyses for genotyping arrays, the multi-chip genotype calling algorithm RLMM was developed. RLMM is based on a robustly fitted, linear model that employs Mahalanobis distance for classification [40]. RLMM achieved a higher call rate than DM. With the release of the Mapping 500 K SNP array set, Affymetrix extended the RLMM model to BRLMM by adding a Bayesian step that provided improved estimates of cluster centers and variances. The DM and GEL algorithms operate on a single chip, while all others use multiple chips to call genotypes.

High call rate and accuracy of genotype calling are important and essential issues for success of GWAS, since errors introduced in the genotypes by calling algorithms can inflate false associations and may lose true associations between genotype and phenotype. Each of the algorithms was reported to have a high successful call rate and accuracy, or more precisely, high concordance with genotypes determined by the International HapMap Consortium on the HapMap samples. With the exception of DM and GEL, the algorithms require data from multiple chips (i.e., a batch) to make genotype calls. A GWAS usually involves analyses of thousands of samples that generate thousands of raw data files (i.e., CEL files). The raw data file for one sample (two CEL files for Affymetrix Mapping 500 K array set: one from Nsp-digested genomic DNA and one from Sty-digested DNA) is about 130 MB in size. Computer memory (RAM) limits make it unfeasible to analyze all CEL files in a GWAS in one single batch on a single computer. The samples are, therefore, divided into many batches for genotype calling. Affymetrix suggests 40 to 96 CEL files for a batch for the BRLMM method. To date, the effects on genotype calls caused (potentially) by changing the number and specific combinations of CEL files in batches and propagation of the effects to the downstream association analysis have not been investigated.

Since BRLMM is recommended by Affymetrix, we analyzed the effect of batch size and composition on the ability of the BRLMM algorithm to consistently call the 270 samples from the International HapMap project.

## Results

### Batch size effect

Batch size effect was assessed by comparing the genotypes called from BS1, BS2, and BS3 (see *Methods*) for call rate and concordance. The overall call rates, defined as the proportion of successful calls to the total number of calls (successful calls plus missing calls) for BS1, BS2, and BS3 were 99.48%, 99.50%, and 99.49%, respectively. However, overall call rates are not informative enough to assess the distribution of missed calls on the chip. Batch size effect on genotype calling rates are best compared using one-against-one comparisons of distributions of call rates on individual samples and SNPs. These distributions were calculated from data of samples and SNPs generated from the calling results of the experiments with three batch sizes (BS1, BS2, and BS3).

**Figure 1:**
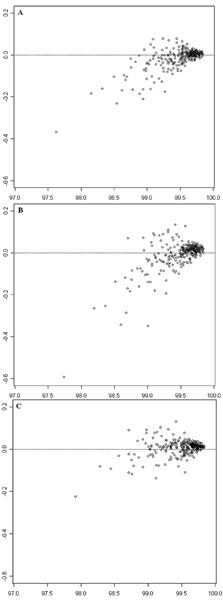
**MA-like plots for comparing call rates of samples between two experiments with different batch sizes**. The empty circles depict the 270 samples. The x-axes represent average call rates of individual samples in two experiments with different batch sizes. The horizontal dotted lines indicate where values of the expected call rates are the same in the two compared experiments. **A**: Comparison between BS1 and BS2. The y-axis represents call rate in BS1 – call rate in BS2. **B**: Comparison between BS1 and BS3. The y-axis represents call rate in BS1 – call rate in BS3. **C**: Comparison between BS2 and BS3. The y-axis represents call rate in BS2 – call rate in BS3.

The comparison of call rates of samples using MA-like plots is shown in Figure 1. The average call rate of two genotype calling results (x-axis) from experiments with two different batch sizes were plotted against the difference of call rates between the two experiments (large batch size – small batch size; y-axis). The horizontal dotted lines at y = 0 represent the expected locations of samples if the missing calls on each sample were exactly the same in the two experiments. Data points above this line are the samples having fewer missing calls (i.e., higher call rate) in the experiment with the larger batch size than in the experiment with the smaller batch size. Data points beneath this line indicate samples having fewer missing calls in the experiment with smaller batch size than in the experiment with the larger batch size. The perpendicular distance from a data point to this line is the difference in call rate of a sample between the two experiments. Figure 1A compares the results of BS1 with BS2; 1B compares the results of BS1 with BS3; and 1C compares the results of BS2 with BS3. Data points at lower average call rates are more distant from the calculated equivalent call rate (dotted line) than the data points at higher average call rates. Thus, batch size affected lower call rates more severely than higher call rates. Furthermore, data points in Figure 1B (BS1 versus BS3) are farther away from the dotted line when compared with the data points in Figure 1A (BS1 versus BS2), which, in turn, were farther away from the dotted line when compared with Figure 1C (BS2 versus BS3). The values of $\bar{D}$ (see *Methods*) were 0.0304, 0.0416, and 0.0257 for comparisons shown in Figure 1A, B, and 1C, respectively, that are related to the corresponding differences of batch sizes of the compared experiments, 45 (90 – 45), 60 (90 – 30), and 15 (45 – 30). The p-values for comparisons in Figure 1A, B, and 1C are 1.736 × 10^-6^, 0.0296, and 0.0116, respectively, indicating that call rates on samples between calling batch sizes are statistically different.

**Figure 2:**
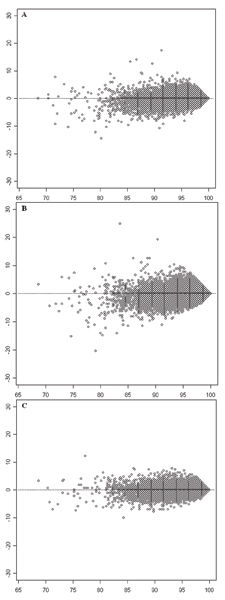
**MA-like plots for comparing call rates of SNPs between two experiments with different batch sizes**. The empty circles depict 500,568 SNPs. The x-axes represent average call rates of individual SNPs in two experiments with different batch sizes. The horizontal dotted lines indicate the expected locations of SNPs where the call rates in the two compared experiments were exactly same. **A**: Comparison between BS1 and BS2. The y-axis represents call rate in BS1 – call rate in BS2. **B**: Comparison between BS1 and BS3. The y-axis represents call rate in BS1 – call rate in BS3. **C**: Comparison between BS2 and BS3. The y-axis represents call rate in BS2 – call rate in BS3.

The comparisons of the call rates for individual SNPs are depicted by MA-like plots in Figure 2. Figure 2A compares the results of BS1 with BS2; 2B compares the results of BS1 with BS3; and 2C compares the results of BS2 with BS3. The trend is similar to that observed in Figure 1 that batch size affected lower call rates more severely than higher call rates for individual SNPs. The $\bar{D}$ values were calculated to be 0.1563, 0.1982, and 0.1467 for the comparisons shown in Figure 2A, B, and 2C, respectively. They were positively correlated with the differences of batch sizes of the compared experiments, 45, 60, and 15, respectively. The p-values for comparisons in Figure 2A, B, and 2C are 2.2 × 10^-16^, indicating that the difference of call rates on SNPs between calling batch sizes are statistically significant.

Comparing call rates in experiments with different batch sizes can only assess the batch size effect on missing calls. Since three genotypes (homozygote, heterozygote, and variant homozygote) are possible for a genotype call, we determined the effect of batch size on the ability to consistently call the genotype. To evaluate the batch size effect on successful calls, concordance of successful genotype calls between experiments with different batch sizes was analyzed (Table 1). Batch size affected successful genotype calls since the concordances were not 100% and heterozygous genotype concordances were more affected than homozygous genotype concordances. The largest difference in batch size (60, BS1 versus BS3) led to the lowest concordances (99.986% overall concordance). However, the concordances for BS2 versus BS3 were slightly lower than for BS1 versus BS2, even though the difference of batch sizes for BS2 versus BS3 (45 – 30 = 15) is smaller than that for BS1 versus BS2 (90 – 45 = 45). This result is likely due to the relatively large difference in the number of arrays in the batch (BS1 = 90 arrays and BS3 = 30 arrays). High concordance of genotype calls depends on the difference between batch sizes as well as the actual batch sizes themselves.

**Table 1:** Concordance of calls between batch sizes

Comparison		BS1 *vs *BS2	BS1 *vs *BS3	BS2 *vs *BS3
Successful Calls for Both	SNPs	134258764	134187584	134265847
	%	99.338	99.285	99.343
Concordant Calls (All)	SNPs	134248899	134187584	134253973
	%	99.993	99.986	99.991
Concordant Calls (Hom)	SNPs	98179772	98136394	98204063
	%	99.997	99.993	99.995
Concordant Calls (Het)	SNPs	36069127	36031744	36049910
	%	99.981	99.964	99.980

Successful calls for both: SNP genotypes successfully called in both of the compared experiments; Concordant calls (All): same genotype called in both of the compared experiments; Concordant calls (Hom): homozygous genotype called in both of the compared experiments; Concordant calls (Het): heterozygous genotype called in both of the compared experiments.

### Batch composition effect

The overall call rate based on all CEL files of the 270 HapMap samples for BC1, BC2, and BC3 (see *Methods*) were 99.48%, 99.43%, and 99.41%, respectively. The genetic homogeneity of the batches in BC1 (samples from 1 population group) is higher than that of BC2 (samples from 2 population groups) which, in turn, is higher than that of BC3 (samples from 3 population groups). The batch sizes were the same for all of the three experiments. Thus, higher call rates were obtained when genotype calling was conducted with samples of higher genetic homogeneity. The effect of batch homogeneity was relatively minor by this measure. Because the distribution of missing calls on samples and SNPs was more informative for assessing batch effect in our first experiments (BS studies), we examined the distribution of call rates in the BC experiments.

**Figure 3:**
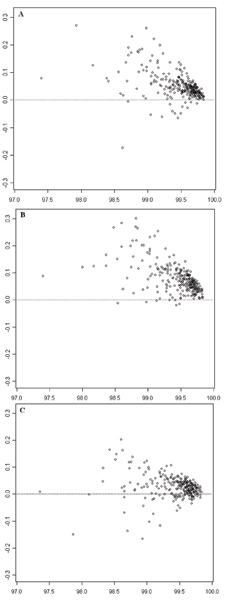
**MA-like plots for comparing call rates of samples between two experiments with different batch compositions**. The empty circles depict the 270 samples. The x-axes represent average call rates of individual samples in two experiments with different batch compositions. The horizontal dotted lines indicate the expected locations of samples where the call rates in the two compared experiments were exact same. **A**: Comparison between BC1 and BC2. The y-axis represents call rate in BC1 – call rate in BC2. **B**: Comparison between BC1 and BC3. The y-axis represents call rate in BC1 – call rate in BC3. **C**: Comparison between BC2 and BC3. The y-axis represents call rate in BC2 – call rate in BC3.

The comparisons of call rates on samples are depicted by MA-like plots (Figure 3). Figure 3A compares the results of BC1 with BC2; 3B compares the results of BC1 with BC3; and 3C compares the results of BC2 with BC3. It can be seen that most of the data points are above the dotted lines, indicating fewer missing genotypes (i.e., higher call rate) when samples in batches are of higher genetic homogeneity. Batch composition had a larger effect when the call rate was lower. Moreover, the level of batch composition effects was related to differences in the genetic homogeneity of samples in the compared batch compositions. We quantified genetic homogeneity as $GH=\frac{1}{n}$, where n is number of population groups of samples in a batch composition. The values of *GH *are 1, 0.5 and 0.33 for BC1, BC2, and BC3, respectively. The $\bar{D}$ values of the comparisons in Figure 3A, B, and 3C are 0.0552, 0.0774, and 0.0373, respectively. These values are positively correlated with the corresponding *GH *differences between the compared experiments, (1 – 0.5 = 0.5), (1 – 0.33 = 0.67), and (0.5 – 0.33 = 0.17). The p-values for all comparisons are 2.2 × 10^-16^. Therefore, the call rates on samples between calling batch compositions are statistically different.

**Figure 4:**
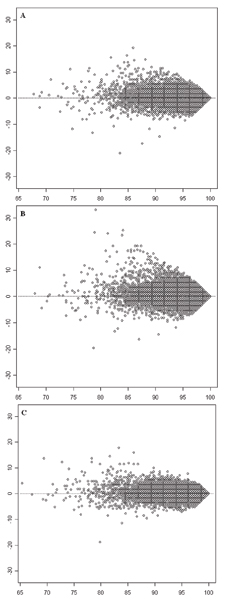
**MA-like plots for comparing call rates of SNPs between two experiments with different batch compositions**. The empty circles depict 500,568 SNPs. The x-axes represent average call rates of individual SNPs in two experiments with different batch compositions. The horizontal dotted lines indicate the expected locations of SNPs where the call rates in the two compared experiments were exactly same. **A**: Comparison between BC1 and BC2. The y-axis represents call rate in BC1 – call rate in BC2. **B**: Comparison between BC1 and BC3. The y-axis represents call rate in BC1 – call rate in BC3. **C**: Comparison between BC2 and BC3. The y-axis represents call rate in BC2 – call rate in BC3.

The comparisons of call rates on SNPs for BC1 versus BC2, BC1 versus BC3, and BC2 versus BC3 are shown in Figure 4A, B, and 4C, respectively. Data points at lower average call rate were farther away from the dotted line than the data points at higher average call rate; that is, batch composition affected SNPs with lower call rates more severely than SNPs with higher call rates. Furthermore, more SNPs are above rather than below the calculated equivalent call rates (dotted line) indicating fewer missing genotypes per SNP (i.e., higher call rate) when samples in calling batches are of higher genetic homogeneity. Moreover, it was further confirmed that the level of batch composition effects was related to differences in genetic homogeneity of samples in the compared batch compositions. The $\bar{D}$ values are 0.2046, 0.2384, and 0.1749 for comparisons shown in Figure 4A, B, and 4C, respectively, that are related to the corresponding *GH *differences between the compared experiments: 0.5, 0.67, and 0.17. The p-values for all comparisons are 2.2 × 10^-16^, confirming that the call rates on SNPs between calling batch compositions are statistically different.

To evaluate batch composition effect on successful genotype calls, concordance of successful genotype calls between experiments with different batch compositions was analyzed (Table 2). Batch composition not only affected the genotype calls but was more pronounced at heterozygous genotypes compared with homozygous genotypes, since the concordance for heterozygous genotype calls were lower than the corresponding concordance for homozygous genotype calls. Moreover, the concordance of successful genotype calls between the compared batch compositions were negatively related to genetic homogeneity differences between the batch compositions. For example, overall concordances were 99.986%, 99.980%, and 99.991% for BC1 versus BC2, BC1 versus BC3, and BC2 versus BC3, respectively. These are in opposite order of the *GH *differences of the compared experiments, that is, 0.5, 0.67, and 0.17 for BC1 versus BC2, BC1 versus BC3, and BC2 versus BC3, respectively.

**Table 2:** Concordance of calls between batch compositions

Comparison		BC1 *vs *BC2	BC1 *vs *BC3	BC2 *vs *BC3
Successful Calls for Both	SNPs	134128046	134063768	134107787
	%	99.241	99.194	99.226
Concordant Calls (All)	SNPs	134109060	134036623	134095792
	%	99.986	99.980	99.991
Concordant Calls (Hom)	SNPs	98050788	97992008	98016851
	%	99.989	99.983	99.993
Concordant Calls (Het)	SNPs	36058272	36044165	36078941
	%	99.977	99.970	99.985

Successful calls for both: genotype successfully called in both of the compared experiments; Concordant calls (All): same genotype called in both of the compared experiments; Concordant calls (Hom): homozygous genotype called in both of the compared experiments; Concordant calls (Het): heterozygous genotype called in both of the compared experiments.

### Quality of the raw data

**Figure 5:**
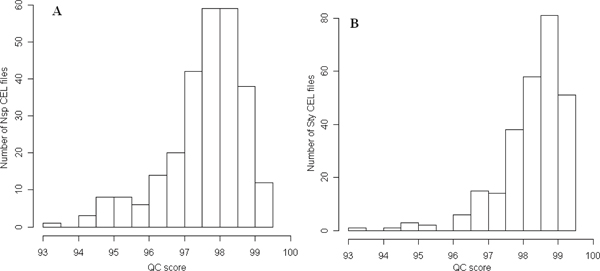
**Histograms of QC confidence scores of Affymetrix Human Mapping 500 K Array Set CEL files of 270 HapMap samples**. The x-axes indicate the QC confidence scores range from 0 to 100. The y-axes represent number of CEL files with QC confidence scores within a window depicted at the x-axes. **A**: Nsp chip CEL files of the 270 HapMap samples. **B**: Sty chip CEL files of the 270 HapMap samples.

The quality of the raw data is important for comparative analyses and interpretation. The QC scores of the 270 Nsp CEL files and of the 270 Sty chip CEL files of the 270 HapMap samples were calculated using DM (Figure 5A and 5B, respectively). The average QC scores for Nsp and Sty CEL files are 97.58 and 98.26, respectively. The lowest QC scores for Nsp and Sty CEL files are 93.49 and 93.18, respectively. The Affymetrix default QC cut-off score is 93. Therefore, we confirmed high QC of the raw data and used all CEL files of 270 HapMap samples in our study.

### Propagation of batch effect to significantly associated SNPs

The objective of a GWAS is to identify the genetic markers associated with a specific phenotypic trait. It is critical to assess whether and how the batch effect propagates to the significant SNPs identified in the downstream association analysis. Three case-control based association analyses were conducted for each of the calling results with different batch sizes and compositions to assess the propagation of batch effect in genotype calling to the significantly associated SNPs (see *Methods*).

After removal of low quality SNPs by quality control assessment, each of the three population groups (European, Asian, and African) was set as "case" while the other two groups were set as "control". Association analyses were conducted to identify SNPs that can differentiate the "case" group from the "control" group. Different lists of SNPs significantly associated with a same population group, identified using the genotype calling results with different batch sizes and compositions, were compared using Venn diagram.

**Figure 6:**
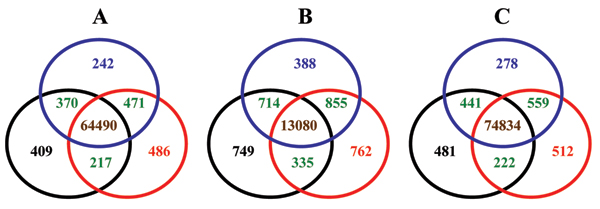
**Venn diagrams for comparisons of the significantly associated SNPs identified using the genotype calling results with different calling batch sizes**. The numbers in circles are the significantly associated SNPs identified in association analyses using calling results from different batch sizes: black circles for BS1, blue circles for BS2, and red circles for BS3. Numbers in brown represent the associated SNPs shared by all three batch sizes, numbers in green represent the associated SNPs shared only by two batch sizes, and the numbers in other colors are the associated SNPs identified only by the corresponding batch sizes. **A**: The association analyses results for European versus others. **B**: The association analyses results for African versus others. **C**: The association analyses results for Asian versus others.

The comparisons of the significantly associated SNPs obtained from calling results with different batch sizes are given in Figure 6. The significantly associated SNPs from BS1 are in black circles, from BS2 in blue circles, and from BS3 in red circles. Number of significantly associated SNPs common in all three batch sizes is in brown, shared only by two batch sizes in green. The association analyses results for European versus others are depicted in Figure 6A, for African versus others in 6B, and for Asian versus others in 6C.

It is clear that the batch size effect on genotype calling propagated into the downstream association analyses. Moreover, it was observed that the larger the differences between two batch sizes, the fewer the significantly associated SNPs shared by the two batch sizes. For example, there were 471, 370, and 217 significantly associated SNPs shared only by BS2 and BS3, by BS1 and BS2, and by BS1 and BS3 for the association analyses with European as "case", respectively, that are negatively related to the corresponding differences of batch sizes: 15, 45, and 60. Same trends were observed for the association analyses with African as "case" and with Asian as "case".

**Figure 7:**
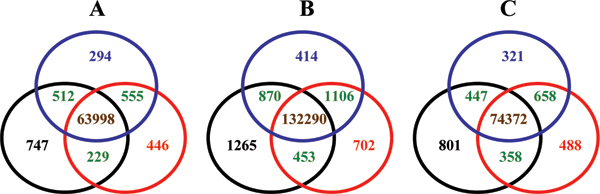
**Venn diagrams for comparisons of the significantly associated SNPs identified using the genotype calling results with different calling batch compositions**. The numbers in circles are the significantly associated SNPs identified in association analyses using calling results from different batch compositions: black circles for BC1, blue circles for BC2, and red circles for BC3. Numbers in brown represent the associated SNPs shared by all three batch compositions, numbers in green represent the associated SNPs shared only by two batch compositions, and the numbers in other colors are the associated SNPs identified only by the corresponding batch compositions. **A**: The association analyses results for European versus others. **B**: The association analyses results for African versus others. **C**: The association analyses results for Asian versus others.

Figure 7 compares the lists of significantly associated SNPs obtained using the genotypes called by the three batch compositions. The significantly associated SNPs from BC1 are in black circles, from BC2 in blue circles, and from BC3 in red circles. Number of significantly associated SNPs common in all three compositions is in brown, shared only by two compositions in green. Association analyses results for European versus others are depicted in Figure 7A, for African versus others in 7B, and for Asian versus others in 7C.

The Venn diagrams demonstrated that for a same "case-control" setting different lists of significantly associated SNPs were identified by the same statistical test (Chi^2 ^test) using the genotype calling results from different batch compositions. Therefore, the batch composition effect on genotype calling propagated to the significantly associated SNPs. Moreover, it was observed that the larger the difference of genetic homogeneity between two batch compositions, the fewer the significantly associated SNPs shared by the two batch compositions. For example, there were 555, 512, and 229 significantly associated SNPs shared only by BC2 and BC3, by BC1 and BC2, and by BC1 and BC3, respectively, for the association analyses with European as "case". The numbers are negatively related to the corresponding differences of genetic homogeneity in the batch compositions: 0.17, 0.5, and 0.67. Same trends were observed for the association analyses with African as "case" and with Asian as "case".

## Discussion

GWAS is increasingly used to identify loci containing genetic variants associated with common diseases and drug responses. The number of SNPs interrogated in a GWAS has grown from thousands to millions; for example, the newest Affymetrix SNPs array 6.0 contains ~2 million probe sets. At the same time, the allele frequency difference of disease-associated or drug-associated SNPs is usually very small. Therefore, a very small error introduced in genotypes by genotype calling algorithms may result in inflated false associations between genotype and phenotype in the downstream association analysis. Reproducibility and robustness are as important to genotype calling as is the accuracy and call rate that are usually used to evaluate performance of genotype calling algorithms. As most genotype calling algorithms are based on multiple chips, and genotype calling for a GWAS is usually conducted in many batches, reproducibility and robustness of multi-chip calling algorithms under different batch sizes and compositions are important variables. Statistical tests of these parameters would increase the confidence for associated SNPs identified in downstream association analysis.

A heterozygous genotype carries a rare allele. Therefore, the robustness of calling heterozygous reduces false positive associations and the chance of missing true associations. Our studies revealed that both batch size and composition affected genotype calling results, especially for heterozygous genotype calling. It was also demonstrated that batch effect propagates to the downstream association analysis. Genotype calling algorithms that eliminate or reduce batch effects but maintain high call rates and accuracy are preferred for GWAS.

BRLMM first derives an initial guess for each SNP's genotype using the DM algorithm and then analyzes across SNPs to identify cases of non-monomorphism. This subset of non-monomorphism SNPs is then used to estimate a prior distribution on cluster centers and variance-covariance matrices. This subset of SNP genotypes is revisited and the clusters and variances of the initial genotype guesses are combined with the prior information of the SNP in an ad-hoc Bayesian procedure to derive a posterior estimate of cluster centers and variances. All SNPs in a chip are called according to their Mahalanobis distances from the three cluster centers and confidence scores are assigned to the calls. With default settings, BRLMM randomly picks 10,000 SNPs to estimate cluster centers and variances. But the number of non-monomorphism SNPs used to estimate the prior distribution on cluster centers and variance-covariance matrices varies with changing number of CEL files and changing composition of CEL files in the calling batches. Batch size effect and batch composition effect alter these estimates of prior distribution and variance-covariance matrices. The effect of altering the number of non-monomorphism SNPs was confirmed when using the BRLMM calling algorithm by varying the batch size and composition. The average number of non-monomorphism SNPs used to estimate the prior distributions are 5468 (Nsp) and 5422 (Sty), 4356 (Nsp) and 4358 (Sty), and 3612 (Nsp) and 3618 (Sty) for calling batches in BS1, BS2, and BS3, respectively. The difference of batch sizes is related to the difference of numbers of non-monomorphism SNPs used to estimate the prior distribution which is, in turn, related to the difference of genotype calling results. The average number of non-monomorphism SNPs used to estimate the prior distribution are 5468 (Nsp) and 5422 (Sty), 6399 (Nsp) and 6308 (Sty), and 6788 (Nsp) and 6688 (Sty) for calling batches in BC1, BC2, and BC3, respectively. Differences in genetic homogeneity of samples are related to differences in the numbers of non-monomorphism SNPs used to estimate the prior which, in turn, is related to the difference of genotype calling results.

## Conclusion

As demonstrated above, both batch size and batch composition affect genotype calling results of GWAS using the BRLMM algorithm. The larger the difference of batch sizes, the larger the effect. When the samples in the calling batches are more homogenous, more concordant genotypes are called. Batch effect propagates to the downstream association analysis and makes the significantly associated SNPs identified inconsistent. Therefore, we suggest from our studies that the same or larger batch sizes should be used to make genotype calls for GWAS and homogenous samples should be put into the same batches.

## Methods

### Raw data

The raw data (CEL files) from the Affymetrix GeneChip Human Mapping 500 K array set of the 270 HapMap samples were downloaded from the International HapMap project website . The CEL file format was described on Affymetrix's developer pages . The file name indicated the population code (CEU/YRI/CHB+JPT), the sample identifier (e.g., NA12345), followed by the Affymetrix array type (based on restriction enzyme name: Nsp or Sty). Three population groups composed the data sets and each group contained 90 samples: CEU had 90 samples from Utah residents with ancestry from northern and western Europe (termed as European in this paper); CHB+JPT had 45 samples from Han Chinese in Beijing, China, and 45 samples from Japanese in Tokyo, Japan (termed as Asian in this paper); YRI had 90 samples from Yoruba in Ibadan, Nigeria (termed as African in this paper).

### Quality of the raw data

The quality of the raw data from the Affymetrix Human Mapping 500 K array set was assessed using DM [39] before genotype calling by BRLMM. DM is a single array based algorithm; it processes one CEL file at a time in a multiple CEL file batch and statistically assesses experimental qualities with a numerical score between 0 and 100. A high QC (quality control) number means high quality of the experiment (CEL file).

### Genotype calling by BRLMM

All experiments of genotype calling by BRLMM reported in this paper were conducted using apt-probeset-genotype of Affymetrix Power Tools 1.8.5. Affymetrix Power Tools (APT) contains a set of cross-platform command line programs that implement algorithms for analyzing and working with Affymetrix GeneChip^® ^arrays. These programs are available on the Affymetrix website . APT programs are intended for "power users" who prefer programs that can be utilized in scripting environments and are sophisticated enough to handle the complexity of extra features and functionality. The function of apt-probeset-genotype in APT is an application for making genotype calls using SNP Arrays (100 K, 500 K, Genome-Wide SNP Arrays 5.0 and 6.0). BRLMM is one of the genotype calling algorithms implemented in this function, and enables many parameters to be changed by a user. For the studies reported here, all the parameters, except as noted in the narrative were set to the default values recommended by Affymetrix. The chip description files (cdf) for both Nsp and Sty chips of the Mapping 500 K array set, as well as files for defining SNPs on chromosome X, were also used before genotype calling. They were downloaded from Affymetrix website. Nsp and Sty CEL files were genotype-called separately.

### Batch size experiments

Three experiments were designed and conducted in order to assess the effect of batch size. In the first experiment (BS1), the 270 HapMap samples were divided into three batches based on their population groups: 90 Europeans, 90 Asians, and 90 Africans. The genotypes were called separately by BRLMM using the default parameter setting suggested by Affymetrix (CEL files from Nsp and CEL files from Sty were analyzed separately). Genotype calling results on Nsp files and on Sty files of the three batches in this experiment were then merged for comparison with results of other experiments with different batch sizes. The second experiment (BS2) used a batch size of 45 samples. Genotypes were called from the CEL files from 90 European samples in two batches, each with 45 CEL files using BRLMM with the same parameter settings as in the first experiment. The procedure was repeated for the Asian and African samples. In the third experiment (BS3), the batch size was 30 samples from each population groups.

### Batch composition experiments

The selection of samples (CEL files) to place in each batch can also be anticipated to alter genotyping call rates. The term batch composition effect is used here to denote the selected arrays within batches. BRLMM was used with default parameter settings and the CEL files of 270 HapMap samples to test batch composition effects. In the first experiment (BC1), the 270 samples were placed in three batches. One batch contained 90 samples from the same population group, Europeans, Asians, or Africans. In the second experiment (BC2), the 90 samples in each of the three population groups were evenly divided into two subgroups with each subgroup having 45 unique samples. Genotype calling was then conducted in three batches with composition of: (i) subgroup 1 of Europeans + subgroup 1 of Asians, (ii) subgroup 2 of Europeans + subgroup 1 of Africans, and (iii) subgroup 2 of Africans + subgroup 2 of Asians. In the third experiment (BC3), the 90 samples in each of the three population groups were evenly divided into three subgroups with each subgroup having 30 unique samples. Genotype calling was then conducted in three batches with composition of: (i) subgroup 1 of Europeans + subgroup 1 of Asians + subgroup 1 of Africans, (ii) subgroup 2 of Europeans + subgroup 2 of Asians + subgroup 2 of Africans, and (iii) subgroup 3 of Europeans + subgroup 3 of Asians + subgroup 3 of Africans. In each of the three experiments, genotype calling results of the three batches were merged together before conducting the comparisons.

### Comparing genotype calling results

In each of the experiments reported here, the genotype calling results by BRLMM from different calling batches were first merged using a set of in-house programs written in C++. When merging the calling results, genotypes of SNPs in Nsp and Sty chips of the same samples were merged followed by assembling together all genotypes of all of the 270 HapMap samples. Thereafter, overall call rates for each of the experiments, call rates of individual samples and SNPs in each of the experiments, and concordant calls between experiments were calculated and exported as tab-delimited text files using the in-house programs written in C++. Comparison of calling results was done using the R package.

Paired two samples t-test in R package (t.test) was used to statistically test the alternative hypothesis that call rates on samples or SNPs between two calling experiments are different.

To quantify batch effect, average absolute differences in call rates were calculated for the comparisons using formula (1).

(1) $$\bar{D}=\frac{\sum_{i=1}^{N}\left|CR_{i}^{1}-CR_{i}^{2}\right|}{N},$$

where $CR_{i}^{1}$ and $CR_{1}^{2}$ are call rates of experiments 1 and 2 of sample i or SNP i, respectively; N is the total number of samples (in this case, 270) or SNPs (in this case, 500,668 which includes 50 QC probe sets in both Nsp and Sty chips).

### Association analysis

In order to study the propagation of batch effect to the significantly associated SNPs, all genotype calling results of the raw data of 270 HapMap samples using BRLMM with different batch sizes and compositions were analyzed using Chi^2 ^statistics test for associations between the SNPs and the case-control settings.

Prior to association analysis, quality control (QC) of the calling results was conducted to remove markers and samples with low quality. For each of the calling results, call rate of 90% was used to remove SNPs and samples. Minor allele frequency was used to filter SNPs and its cut-off was set to 0.01. Departure from Hardy-Weinberg equilibrium (HWE) was check for all SNPs. The p-value of Chi^2 ^test for Hardy-Weinberg equilibrium was calculated for all SNPs at first and then the p-values were adjusted for multiple tests using Benjamini and Hochberg false discovery rate (FDR) [41]. FDR of 0.01 was set as the cut-off for HWE test. There were no samples removed because of low quality. 54942 (10.97%) to 55496 (11.084%) SNPs were removed in the QC, mainly because of departure from HWE.

To mimic "case-control" in GWAS, for each of the genotype calling results, each of the three population groups (European, African, and Asian) was assigned as "case" while the other two as "control" to form a data set for association analysis for identifying the SNPs significantly associated with the "case" population group.

In the association analyses, a 2 × 3 contingency table was generated for each SNP and a case-control setting. Then Chi^2 ^statistics test was applied on the contingency table to calculate a p-value for measuring the statistical significance of the association between the testing SNP and the corresponding case-control setting. After raw p-values for all SNPs in a data set were calculated, Bonferroni correction was applied to adjust the raw p-values. Lastly, a criterion of Bonferroni-corrected p-value less than 0.01 was used to identify the significantly associated SNPs.

## Competing interests

The authors declare that they have no competing interests.

## Authors' contributions

HH coordinated the project, designed the experiments, conducted the genotype calling and association analysis, compared the calling results using R package, and wrote the manuscript. ZS wrote all of the in-house C++ programs, and involved discussions on the experiments and analysis of the calling results. WG calculated all of the call rates and concordant calls and involved discussions on the experiments and analysis of the calling results. LS, RP, JK, JCF, and WT involved discussions on designing the experiments and analysis and assisted the writing manuscript. HF, JX, JC, and TH involved discussions on experimental design and data analysis. All authors read and approved the manuscript.
